# Different spreading dynamics throughout Germany during the second wave of the COVID-19 pandemic: a time series study based on national surveillance data

**DOI:** 10.1016/j.lanepe.2021.100151

**Published:** 2021-06-27

**Authors:** Andreas Schuppert, Katja Polotzek, Jochen Schmitt, Reinhard Busse, Jens Karschau, Christian Karagiannidis

**Affiliations:** a Institute for Computational Biomedicine, JRC for Computational Biomedicine RWTH Aachen University, University Hospital Aachen; b Centre for Evidence-based Healthcare, University Hospital Carl Gustav Carus and Carl Gustav Carus Faculty of Medicine, Technische Universität Dresden; c TU Berlin, Department of Health Care Management, Technische Universität Berlin, Berlin, Germany; d Department of Pneumology and Critical Care Medicine, Cologne-Merheim Hospital, ARDS and ECMO centre, Kliniken der Stadt Köln, Witten/Herdecke University Hospital, Cologne, Germany

## Abstract

**Background:**

The second wave of the COVID-19 pandemic led to substantial differences in incidence rates across Germany.

**Methods:**

Assumption-free k-nearest neighbour clustering from the principal component analysis of weekly incidence rates of German counties groups similar spreading behaviour. Different spreading dynamics was analysed by the derivative plots of the temporal evolution of tuples [x(t),x’(t)] of weekly incidence rates and their derivatives. The effectiveness of the different shutdown measures in Germany during the second wave is assessed by the difference of weekly incidences before and after the respective time periods.

**Findings:**

The implementation of non-pharmaceutical interventions of different extents resulted in four distinct time periods of complex, spatially diverse, and age-related spreading patterns during the second wave of the COVID-19 pandemic in Germany. Clustering gave three regions of coincident spreading characteristics. October 2020 showed a nationwide exponential growth of weekly incidence rates with a doubling time of 10 days. A partial shutdown during November 2020 decreased the overall infection rates by 20–40% with a plateau-like behaviour in northern and southwestern Germany. The eastern parts exhibited a further near-linear growth by 30–80%. Allover the incidence rates among people above 60 years still increased by 15–35% during partial shutdown measures. Only an extended shutdown led to a substantial decrease in incidence rates. These measures decreased the numbers among all age groups and in all regions by 15–45%. This decline until January 2021 was about -1•25 times the October 2020 growth rates with a strong correlation of -0•96.

**Interpretation:**

Three regional groups with different dynamics and different degrees of effectiveness of the applied measures were identified. The partial shutdown was moderately effective and at most stopped the exponential growth, but the spread remained partly plateau-like and regionally continued to grow in a nearly linear fashion. Only the extended shutdown reversed the linear growth.

**Funding:**

Institutional support and physical resources were provided by the University Witten/ Herdecke and Kliniken der Stadt Köln, German ministry of education and research ‘Netzwerk Universitätsmedizin’ (NUM), egePan Unimed (01KX2021).

## 1 Introduction

**Figure 1:**
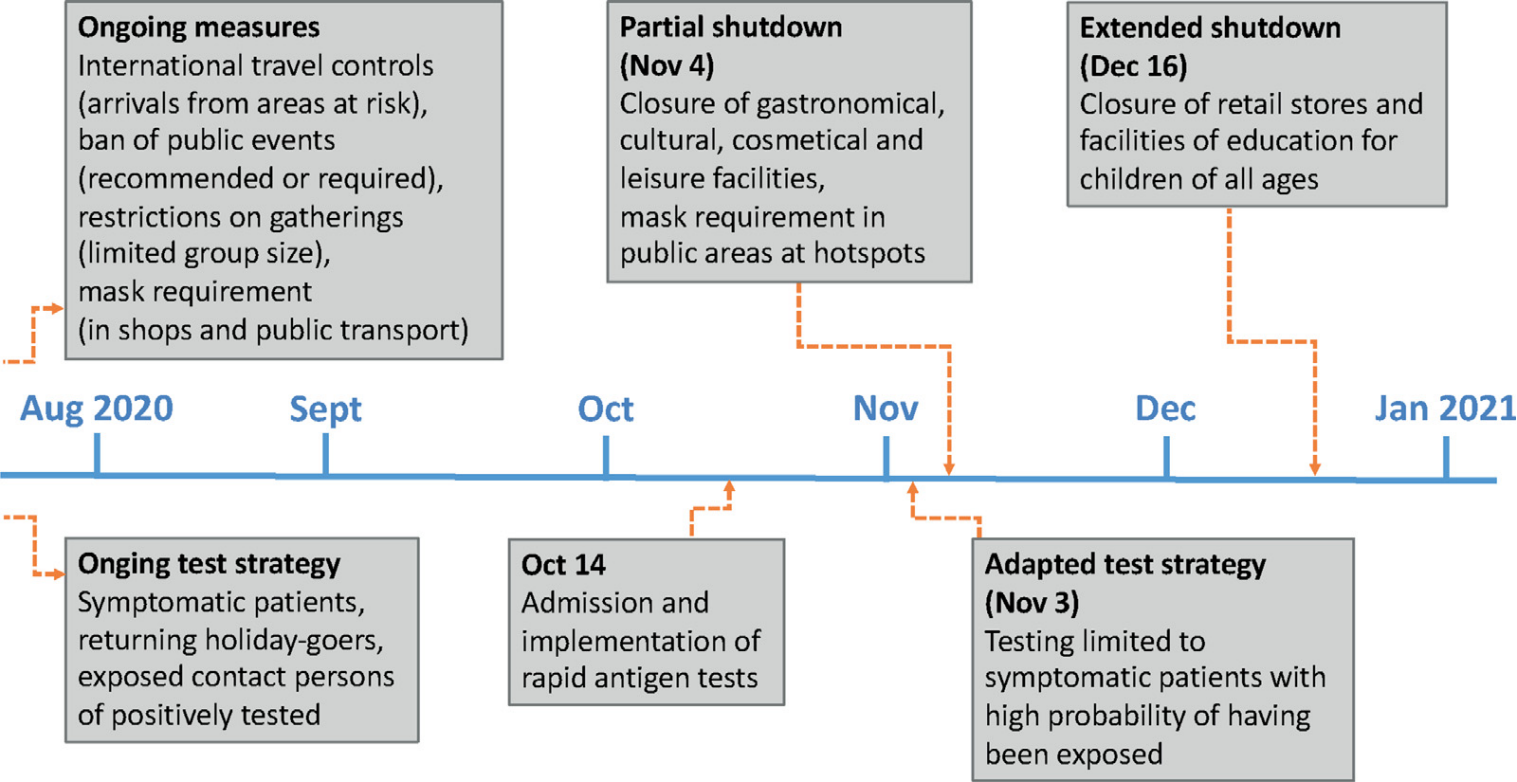
Timeline of German governmental response to emerging incidences during the second wave of the COVID-19 pandemic. The time bar indicates the first day of the months from August 2020 to January 2021. ‘Ongoing’ denotes approaches implemented during the first wave in spring 2020 and valid ever since, such as mask requirements or regulations on travel and gatherings. Since 2 November 2020, a modified test strategy deploys testing capacities efficiently by considering only symptomatic patients with a high risk of having been exposed to the virus. During winter, test resources were highly challenged otherwise due to the coinciding periods of the flu and common cold. From 4 November 2020, a partial shutdown mainly determined the closure of venues for leisure time activities, such as restaurants and museums, but also of personal hygienics like hairdressing salons. An extended shutdown beginning on 16 December 2020 extended the measures by the closure of schools and retail shops.

The coronavirus disease 2019 (COVID-19) pandemic has affected more than 100 million people worldwide. In Germany, by 15 February 2021, 2•3 million people were tested positive for SARS-CoV-2, resulting in high morbidity and mortality rates [[1], [2], [3], [4], [5]]. The second wave of infections started at the end of September 2020, peaked at 34 000 daily new cases in December 2020, and was still ongoing in March 2021 [6]. During that period the wild type of the virus was still the dominant pathogen, and no vaccination program had yet affected the immunity among the German population. The second wave was characterized by the effects of non-pharmaceutical interventions (NPI), namely first partial (4 November 2020), and then extended (16 December 2020) shutdown measures in Germany (Figure 1, Suppl. Figure 1). It should be noted that from 4 November to 16 December 2020, the daily incidence of SARS-CoV-2 varied greatly from 15/100 000 in northern parts of Germany to 120/100 000 in the eastern regions [27].

Regarding these different incidence rates within one country, it has been recently shown by Thurner et al. [8] that the spreading of infection dynamics in social contact networks (SCNs) exhibits at least two dynamic modes: a mode showing exponential growth of daily infections and a dynamic mode showing linear growth. Linear dynamics in time is expected to dominate the spreading in SCNs below a certain threshold of contacts, whereas in SCNs with high connectivity exponential growth dynamics would dominate. In particular, superspreading events may have a substantial effect on exponential growth [[9], [10], [11], [12], [13]], whereas linear growth is driven by diffusive spreading throughout parts of SCNs with low connectivity [[8], [14], [15]] Hence, shutdown measures prohibiting primarily nodes with high connectivity in SCNs may show poor effectiveness in reducing diffusive spreading [[15], [16], [17]]. While early advances in understanding spreading dynamics have already been done much of this work relies on data of the first wave of the COVID-19 pandemic which in hindsight is scarce compared to the second wave in Europe [[18], [19], [20]].

With regard to substantial differences in incidence rates across Germany and the insufficient effects of partial shutdown measures, we aimed at analysing SARS-CoV-2 infection dynamics with respect to non-uniform modes of growth dynamics associated with the effectiveness of such shutdown measures. In our study we focussed on a data-driven approach for the effects of the two different shutdown measures set in place in Germany throughout the second wave. We analysed the effects of both types of shutdowns on the type of spreading dynamics, as discussed in Thurner et al. on a general scale. Due to lack of data in Germany, a detailed analysis of the restrictions of the person-to-person interactions could not be performed. For characterisation of the spreading phenotypes, we used cluster analysis, a method for model-free automatic community detection, for the identification of a spatial distribution of spreading modes among German counties and analysed the above-mentioned shutdown measures and how they influenced the spreading behaviour. The clustering revealed a dominant spreading pattern for each German state, thereby reflecting the regional organization of the German health care system within 16 administrative regions and allows for time series analyses on federal-state level.

## 2 Methods

### 2.1 Distinction of spreading modes and shutdown effectiveness by time periods

Depending on the aim of our time series analyses, we used different time windows of the reported incidences. Our clustering and derivative plot analyses described below focus on specific characteristics of spreading dynamics, both in growth and shutdown phases. For the effectiveness of NPIs and, in particular, the phase of declining incidence, we consider absolute differences between the beginning and end of the corresponding time windows only. The dynamic phases of spreading were selected according to the NPIs being put into effect (4 November and 16 December 2020) and inflexion point analysis (end of November 2020) of the spreading dynamics.

For the automated clustering of the spreading dynamics at the county level, we considered data between 1 September 2020 (beginning of a nationwide increase in incidences by the end of the summer break in schools) and 20 December 2020 (Christmas holidays). Due to less testing and reporting during the Christmas period and turn of the year 2020, surveillance data is less meaningful and spreading features cannot be studied in detail reliably.

Nevertheless, considering additional data from January 2021 and differences in weekly incidences within the dynamic phases before and after critical system changes by NPIs allows for robust inferences about their effectiveness, despite the missing data in the end of December 2020. To quantify the dynamics between the start and endpoints of the respective dynamic phases, we calculated the means of the incidences within the seven-day start and end periods:

**Table:** 

	Start-period week	End-period week
October growth phase	4 Oct. – 10 Oct. 2020	30 Oct. – 5 Nov. 2020
Partial shutdown phase	30 Oct. – 5 Nov. 2020	24 Nov. – 30 Nov. 2020
December growth phase	24 Nov. – 30 Nov. 2020	16 Dec. – 22 Dec. 2020
Extended shutdown phase	16 Dec. – 22 Dec. 2020	10 Jan. – 16 Jan. 2021

For analysing the dynamics of the incidences across the phases, we used the differences between the respective end periods and start periods. Data was normalized to 100 000 inhabitants.

### 2.2 Clustering of German counties by their infection dynamics

**Figure 2:**
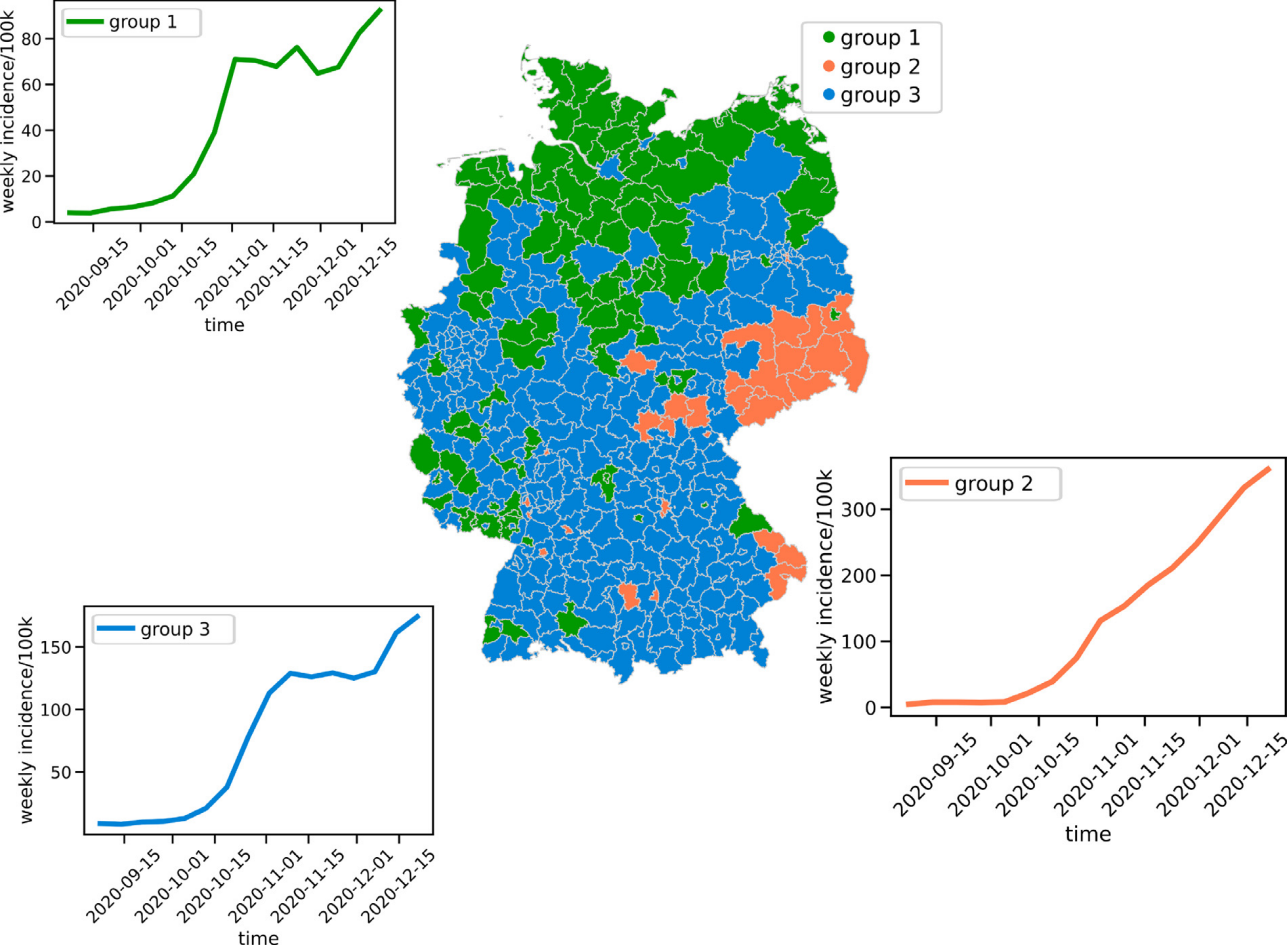
Clustering of spreading patterns. Spreading dynamics were clustered based on their similarity to either of three groups. Without spatial information of the time series alone, these yield three spatially coherent regions. The median of all curves of each group shows the dominating COVID-19 spreading dynamics of these regions. All the curves display an exponentially fast onset of case numbers in October 2020. It could be halted or decelerated in Groups 1 and 3 during November by the partial shutdown but rose up again by December. In Group 2, the partial shutdown only slowed down the overall infection spreading to a non-exponential, but at least linear, increase in incidence numbers per week. In contrary, there was no stopping in Group 2, where daily reported cases further increased continuously.

We used weekly infection incidences of all 412 German counties (Fig. 2, map). This data was normalized by the number of inhabitants per county to yield weekly rates of infections per 100 000 inhabitants. We obtained the dataset from the SurvStat@RKI database of the German Robert Koch Institute (RKI) on 12 March 2021 [21]. The considered time period includes 16 calendar weeks from 1 September 2020 to 20 December 2020.

The infection dynamics were analysed by an all-to-all mapping of county incidence curves. The size of the population is similar among the German counties. This in combination with the normalization per 100 000 inhabitants provides statistical comparability and should be taken into account when adopting our method to other countries. To compare each of the counties with another, we used a ‘dynamic time-warping’ metric with a score value of zero for perfectly matching curves and a value positively greater than zero for more dissimilar curves. Based on this score, each curve was grouped using the k-nearest neighbour method on the first principal components of this heat map. This reduction of the dimension includes three groups in cluster time curves, resulting in each group's median time course as the prototypical curves for infection dynamics (Fig. 2, graphs).

We implemented the grouping in Python 3.8, using the package ‘scikit-learn’ and ‘dtwaidistance’ for implementations of the clustering and dynamic time-warping algorithms, respectively.

### 2.3 Accumulation of infection numbers across counties and federal states

**Figure 3:**
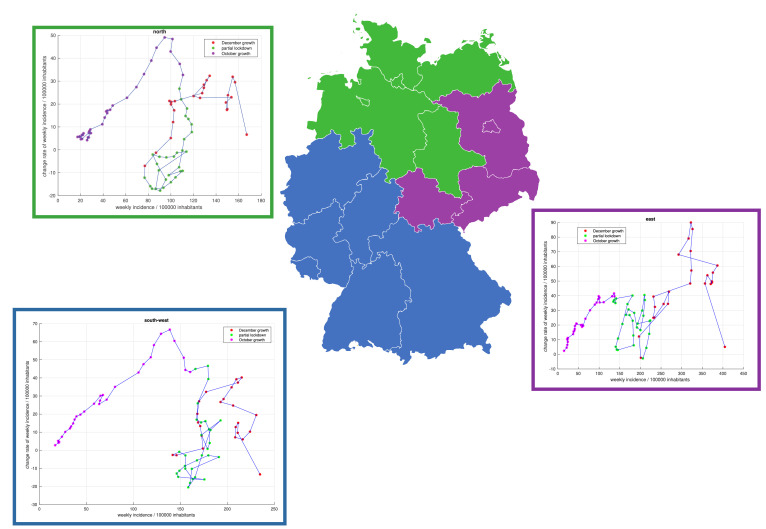
Clustering of states and infection dynamics. The clustering at the county level (cp. Fig. 2) yields three groups of German states with different infection dynamics—the northern (green), southwestern (blue), and eastern (purple) parts of the country. The three derivative graphs draw the cluster's weekly incidences per 100 000 population against the rate of change of the same. Links between single points denote the temporal evolution of these tuples. The straight-line relations, such as depicted for the October growth period (purple), in all plots indicate an exponential temporal evolution of the weekly incidences. In each of the three state clusters during the partial shutdown (green), this exponential growth collapses to non-exponential infection dynamics thereafter.

Clustering on county level provides statistical robustness in the following two directions. Stochastic variability dominates infection numbers on smaller scale such as cities or groups of villages and impedes reliable inference of common spreading dynamics. Summing over incidences at greater state-level, however, might lead to averaging out significant effects or mitigate characteristics of the infection numbers, when the spreading dynamics at county-level differs greatly from one county to another without a dominant overall behaviour. Thus considering regions on the spatial scale of counties ensures clustering by the right dynamical features. Without further assumptions the clustering result reliably implied a prominent pattern of evolution for each of the German states that guided the spreading of the pandemic. The analysis is quantitatively unaffected by the existence of single deviant counties inside a state. The overall spatial coherence of group membership in Figure 2 resembles state clusters in Figure 3, reflecting the federal governance in Germany and each state's organisation of NPIs. Each federal state must implement its pandemic prevention plan for all its counties considering the most badly affected ones.

### 2.4 Characterization of spreading dynamics

An assessment of exponential versus non-exponential growth dynamics (Fig. 3, graphs) used derivative analysis plotting of [x(t), x’(t)] of the weekly incidences and their weekly change rates. Weekly-accumulated incidences smoothen out daily variations induced by the reporting bias exhibiting a strong weekly periodicity. Hence weekly accumulated incidences smooth-out short-term dynamics on the one side but enable assessment of mid-term dynamics without reporting bias. As the weekly variance of the incidences among the three clusters shows almost proportionality to the incidence numbers, a special smoothing algorithm was applied, which is described in the supplementary material. A linear shape of [x(t),x’(t)] within the time interval [t_1_,t_2_] guarantees exponential growth within [t_1_,t_2_]. The slope α of linear regression in a derivative plot of an exponential function is its rate constant and indicates growth or decrease. Using an exponential description with a basis of two implies a doubling time of log(2)/α in the considered temporal unit, which, in our case, is in weeks. Non-linear patterns in a derivative analysis plot indicate non-exponential dynamics. A vanishing slope in the plot associated with a constant derivative x’(t), suggests linear growth or decline at this very rate.

The change rates between start and end of the spreading and shutdown phases were calculated by differences in weekly averages of the original infection data. For pooling of federal states, we normalized the infection rates by the overall population of the state to incidences per 100 000 population.

The dynamics of infection spreading across the spatial clusters and age groups were assessed by means of the differences of weekly reported cases between the end and start of the spreading phases: growth in October, partial shutdown in November, growth again in the first half of December, and extended shutdown starting by 16 December 2020.

Overall, the dynamics of growth phases and shutdowns were assessed across spatial clusters and age groups by means of differences of weekly incidence rates, normalized to 100 000 inhabitants within the respective state cluster. The differences were calculated between and normalized to 100 000 inhabitants. The time course of the age distribution of cases was assessed using the proportion of the respective age cohorts in 10-year intervals without any spatial stratification (Supplemental Figure 4).

All analyses were performed using Matlab, Version R2020a, The MathWorks Inc., Natick, Ma.

### 2.5 Role of the funding source

The funders had no final role in the study design; in the collection, analysis and interpretation of data; in the writing of the report; or in the decision to submit the paper for publication. All researchers listed as authors are independent from the funders and all final decisions about the research were taken by the investigators and were unrestricted.

## 3 Results

### 3.1 Regionally different infection dynamics despite similar shutdown measures

Clustering on dynamic time-warping similarity maps gives three spatial groups of counties with different characteristics in their infection dynamics. A protocurve comprises the median shape of the weekly incidences per 100 000 population for each of the three groups (Fig. 2) and their differences and similarities. Group 1 shows the quantitatively lowest numbers of incidences of all with at most double-digit weekly incidences per 100 000 inhabitants. The growth of weekly reported infections proceeds similarly in Group 3, although it is twice as high. Group 2 exhibits a decisively different pattern of spreading. While in Groups 1 and 3 the partial shutdown measures induced an end of the exponential increase with a plateau of weekly infection numbers during November 2020, in Group 2 the same measures broke the exponential growth of October 2020 but did not prevent the continuing, albeit near-linear, growth of incidences in these regions. All the groups experienced again increasing infection numbers starting from the beginning of December 2020.

The regional location of the counties of the three groups allows for a clustering at the federal state level into the three clusters north, southwest and east (Fig. 3, map) as explained in the last but one method section above. In all the clusters, the growth during October is exponential by the derivative analyses (Fig. 3, graphs). The slope of the linear regressions’ measures about α ≈ 0•5 1/week for each of the state groups, which indicates a doubling time of the weekly incidences of about 10 days. The protocurves in Figure 2 reflect this observation. By the beginning of November 2020, the purely exponential growth ended in all German federal states. Afterwards, a short decrease in the wiggling characterizes the relation between the weekly incidences and their derivatives (Fig. 3, graphs). Between November and December 2020, the behaviour was non-exponential. However, this non-exponential growth was nearly linear. Therefore, for the sake of simplicity, we distinguish exponential from linear growth in the following text. The period of a constant derivative—i.e., a linear shape with slope zero in a derivative plot—would suggest a linear spreading pattern, but it is not explicit from the graphs in Figures 2 and 3. In the northern and southwestern clusters, the partial shutdown yields decreasing and even negative derivatives during November, which is not the case in the eastern group. By December, in each of the three state clusters, the rate of change of weekly incidences became positive again with periods of growing at least or faster than linearly.

### 3.2 Regional and age-dependent impact of shutdown measures

**Figure 4:**
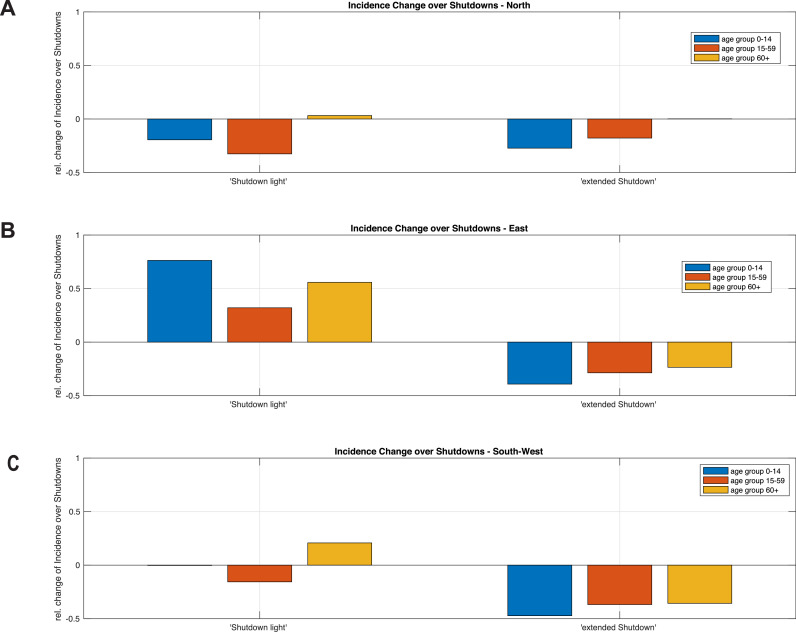
Effectiveness of containment measures. The partial and extended shutdown measures induced different effects on the incidences in different age groups and federal state clusters. The change of the weekly reported new cases is normalized by the first day of each spreading period. In the northern and southwestern clusters, the partial shutdown decreased the numbers among younger age groups. The elderly group, and in the eastern states all age groups, kept rising in the weekly change of cases. Only the extended shutdown decreased the incidence among all age groups and states.

Across all federal states, the working cohort of 15–59 years exhibited highest growth rates during the exponential increase in October 2020 (Suppl. Fig. 2). The crucial difference between the state clusters lies in the effect of the shutdowns in this cohort, and it is quantified by means of relative changes due to the partial shutdown (Fig. 4A-C) and extended shutdown (Fig. 4A-C) measures. While in the northern (Suppl. Fig. 2A) and southwestern (Suppl. Fig. 2C) states incidence rates not only decelerated, but also even decreased, in the eastern states the growth rate remained positive, albeit at reduced levels (Suppl. Fig. 2B). In addition, in all states the growth rate among the elderly population increased during the partial shutdown. Under continued partial shutdown regulations, all states and age groups experienced another onset of increasing infections by the end of November 2020. In the northern and southwestern states, the December 2020 growth phase showed slower growth compared to October 2020 for the younger age groups, whereas similar or even higher absolute growth rates in the elderly cohort (Suppl. Fig. 2A, C). This comparison is prominently expressed in the eastern cluster among all age groups (Suppl. Fig. 2B). Only the extended shutdown measures reduced the infection rate, such that the number of new infections decreased in all age-groups and all states (Suppl. Fig. 2B).

### 3.3 Regional and age-dependent effectiveness of shutdown measures

**Figure 5:**
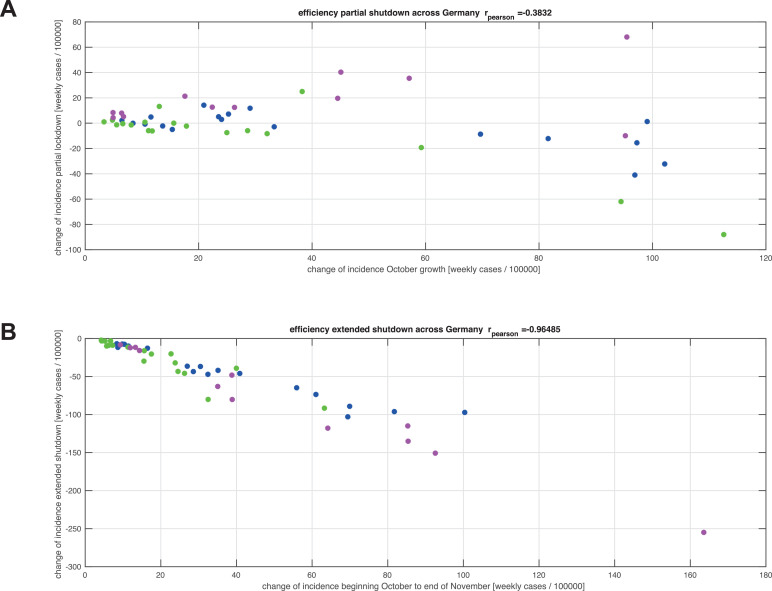
Effectiveness of shutdown measures. Panels A and B correlate the weekly increase of incidences per 100 000 population in October 2020 and the weekly change of incidences per 100 000 population per age group and federal state during the partial and extended shutdown periods, respectively. The colours of the points (green, blue, and purple) correspond to the clusters north, east, and southwest in Figure 3. Positive changes on the y-axis denote increasing numbers of weekly new infections, while negative changes denote decreasing weekly new infections. Panel A shows a weak correlation between October growth and strength of slowing down by the partial shutdown in November. Panel B indicates a strong correlation between the October growth and the decrease during the extended shutdown in December.

In the northern and southwestern clusters, the partial shutdown decreased the weekly reported cases by about 20 – 40% across all age groups (Fig. 4 A-C). In the eastern group, however, we found a further increase by 30 – 80%. Comparing the effectiveness of the partial shutdown, quantified by the decrease in the number of reported cases and the increase in the number of cases throughout October across federal states and age cohorts, we found a weak correlation of r = -0•38 (Fig. 5A).

There is, however, a remarkably strong negative correlation of r = -0•96 among all federal states and age groups between the undamped increase of weekly incidences in October and during the partial shutdown in November compared to the decrease in the same number of incidences in December and mid-January due to extended shutdown regulations (Fig. 5B). The decline of weekly incidences by the extended shutdown exceeds the October/November increase by a factor of about -1•25. Variability in age and location does not affect the relation between October/November spreading and extended shutdown, thereby indicating that the extended shutdown counteracted almost reversibly the spreading modes active in October, which could not be compensated by the partial shutdown in November. This is in clear contrast to the partial shutdown in November where no uniform relation could be found (Fig. 5A).

### 3.4 Test strategy

During the emergence of the second wave, the number of PCR tests doubled from about 800,000 in the beginning of August 2020 to about 1•4 – 1•6 million per week by November 2020 [22]. Since November the number of weekly tests have remained almost constant at this level until the Christmas period (Suppl. Fig. 3). The doubling time of the weekly incidences measured about 10 days, whereas the doubling time of the numbers of tests was about eight times longer than the former. The exponential growth during October cannot be solely attributed to increased numbers of PCR tests. The same applies to the plateau of weekly cases in the clusters north and southwest in November whilst the number of tests was constant. The further increase of cases in the cluster east explains the increase of the positive rate of conducted tests throughout the entire second wave (Suppl. Fig. 3). Supplemental Figure 4 provides further details on the contribution of the different age groups to the positive PCR tests.

## 4 Discussion

The current data demonstrates that there was a nationwide exponential growth throughout Germany in October 2020. A partial shutdown in November 2020 changed this into a spatially diverse and age-related complex spreading pattern, resulting in a strong non-exponential growth dynamic that began in December 2020 and contained by an extended shutdown implemented in mid- December. The cluster analysis of infection dynamics within this time window revealed three distinct types of regionally different dynamic patterns across all four phases, primarily differing in the effect of the partial shutdown ranging from containment in some regions to a near-linear increase, particularly in the east.

Our assumption-free clustering of counties by their spreading dynamics revealed a dominant shape for each of the federal states. This allows for studying infection patterns accumulated over the counties belonging to the federal states (Supplementary Material). However, from a public health perspective, federal states are mainly responsible for political decisions, regulation of healthcare interventions, hospitals, and implementation of all measures. Having a prevalent behaviour across a region under shutdown measures, therefore, reflects the regional organization of political responsibilities by federal states in Germany, although most of the measures were discussed and coordinated between the federal states and the chancellor. Hence, the spatial dynamic clusters were related to the federal states so that a division into north, southwest, and east became apparent. It should be noted that the eastern regions close to the border had a noticeably stronger increase than the other regions, although in western parts of Germany incidences in countries close to the border (i.e. Belgium, France, and the Netherlands) were high in October and November [[23], [24], [25]]. The same accounts for the southerner parts close to the borders of Switzerland and Austria [26]. Therefore, with regard to public health interventions, at least symptom screening at the borders may have an effect on this spreading behaviour, even if we could not quantify the rate of commuters [27].

Interestingly, the partial shutdown introduced in November prevented further exponential growth, dominating in October/November the dynamics in the southwest and north cluster and federal states for the younger age groups. The partial shutdown had, however, only limited influence on the more linear, rather diffuse growth in the east, and, surprisingly, did not reduce infection dynamics in the elderly groups throughout Germany. This near-linear growth was reversed only through the extended shutdown period and was generic; it was, therefore, not limited to individual expansion patterns nor differed among the age groups.

Moreover, we found growth dynamics in October within the elderly age group in the east more prominently than in the rest of the country, followed by growth throughout November which was most prominently in the eastern cluster. However, the relative proportion of younger infected people decreased significantly faster and therefore resulted in a relative increase in the number of older infected patients. This observation is in accordance with the hypothesis that the growth dynamics in the elderly cohort was affected by diffusive growth, whereas the growth dynamics in the younger cohorts throughout October had been driven by exponential growth such as superspreading events. The surprising correlation between the extended shutdown effectiveness and October growth dynamics, which could not be compensated by partial shutdown measures in November (assessed by the difference in the end of November and beginning of October incidences), was not affected by the variation in age and location, thereby indicating the existence of a unique dominating spreading mechanism resulting in a near-linear growth, which can be reversed only by extended shutdown measures. Targeted measures, as implemented throughout the partial shutdown, succeeded in hitting the spreading mechanisms of October by breaking the exponential growth all over Germany. Nonetheless, the measures only achieved a deceleration of the growth in eastern regions to further near-linear spreading and a plateau of constant numbers of new daily incidences in the north and southwest. Both settings resulted in the rising infection numbers by December. Containment resulting in a decrease in daily reported new cases was only achieved by extended wide-ranging shutdown measures in December.

Furthermore, we analysed the effect of test intensity on the age distribution of reported cases. The revised test strategy—i.e. limiting tests to symptomatic patients with a high probability of having the disease—not only led to a higher rate of test-positive cases overall (Suppl. Fig. 3), but also influenced the age composition of test-positive persons. The percentages of younger age cohorts decreased, while those of older age cohorts increased (Suppl. Figs. 4 and 5). Hence, the apparent correlations between time, test positive ratio, and ratio of cases in the various age groups show the tight interdependence between test strategies driven by resources, growth of incidence rates, and age-related stratification. These findings indicate the need for more balanced test strategies to reveal more detailed information on the infection dynamics.

Our findings further demonstrate that containment in older age groups was not sufficient enough to prevent infections, particularly associated with higher mortality rates, than in the younger population, as shown by the corresponding increasing death rates in this timeframe [2]. This age dependency was robust even if differences in test strategy were included. A clear and sustained decrease was only apparent after an extended shutdown in December was introduced.

## 5 Limitations

Our study has certain limitations. First, the precise effect of different targeted shutdown measures could not be identified, such as the impact of closure of schools, theatres, or retail shops on the pandemic spreading, especially among different age groups. Second, the influence of infection dynamics in federal states close to the borders by cross-border traffic and commuters could not be determined in detail. This points out the importance of a pan-European approach, which is of particular importance, since the virus spreads irrespective of any borders [27] There is also no data about adherence to preventive measures that might differ between the different federal states. Last, in the considered period between September 2020 and mid-January 2021, the influence of mutated virus variants and vaccination programmes on the pandemic was still negligible in Germany. The principles of our analysis transfer to future, and meanwhile, different pandemic settings, except for the concrete numbers, were found for the characterization of the spreading dynamics and shutdown efficacy.

## 6 Conclusions

Constant screening of different behavioural patterns, particularly with regard to the new mutants, is essential in public health. The partial shutdown was only moderately effective and at most cuts the exponential growth, but the spread remains partly plateau-like and regionally continued to grow nearly linear. Only the extended shutdown stopped the non-exponential growth, whereby the breaking effect was about 1•25 times stronger than the acceleration of the expansion. Thus, public health interventions targeting super spreading events by partial shutdown measures may cut the exponential growth, but they have no (or little) influence on the more linear and diffusive spreading. Hence, focused enforcement of shutdown measures requires a continuous assessment and adjustment of the measures to target the heterogeneous drivers of spreading dynamics with the optimal effectiveness.

### Research in context

**Evidence before this study** The current COVID-19 pandemic came with an even stronger second wave, beginning in the late summer of 2020 in Europe. In view of the exponential growth of infections during October 2020, Germany first introduced a partial shutdown in the beginning of November 2020, followed by an extended shutdown by mid-December. We performed a search on PubMed on 17 February 2021 with regard to different spreading mechanisms and shutdown measures in different regions. There are currently no regional and age-stratified results on this published data at the time of search.

**Added value of this study** The analysis of spreading dynamics with mathematical methods for time series analysis and pattern recognition methods shows regional differences across German federal states. The analysis uses a two-step approach by quantitatively describing the velocity of COVID-19 under particular measures and clustering regions based on the similarity of their spreading dynamics. Despite a lack of spatial information regarding clustering regions purely based on their incidence time courses, the clustered regions form coherent spatial groups that exert prototypic similar spreading dynamics. We found both exponential and near-linear growth dynamics, indicating non-uniform spreading mechanisms. We found high spatial and age-specific diversity in halting infection dynamics under partial shutdown measures, which has the maximum effect of halting infection spreading in age groups < 20 years and reduced effects in age groups above 60 years. The effect of extended shutdown measures, however, was able to affect all age groups across all states in halting the spreading of SARS-CoV-2 infection and decreased the cases at a rate that is 1•25 times faster than unhindered disease spreading.

**Implications of all available evidence** The methods used here should form a standard tool set for the analysis of infection spreading dynamics. Being able to group regions and to identify the type of spreading dynamics can be used to provide an early warning system to (i) detect and (ii) predict changes in spreading dynamics, and thus, the effectiveness of the applied measure to halt and reverse the infection dynamics.

### Contributors

All the authors conceived the study and its design, had full access to the data, and take responsibility for the integrity of the data and accuracy of the analysis. A.S., K.P., and J.K. organized and entered data. All other contributed to data analyses and data interpretation. C.K., A.S. and K.P. wrote the main draft of the manuscript. All the authors contributed to the final drafting of the manuscript; they also read and approved the final manuscript.

### Declaration of interests

Dr. Karagiannidis reports personal fees from Maquet, personal fees from Xenios, personal fees from Bayer, non-financial support from Speaker of the German register of ICUs, grants from German Ministry of Research and Education, during the conduct of the study. Dr. Schuppert reports grants from Bayer AG, outside the submitted work. Dr. Jens Karschau has nothing to disclose. Dr. Polotzek has nothing to disclose. Dr. Schmitt reports personal fees from Sanofi, Lilly, ALK, Novartis, grants from Sanofi, Pfizer, ALK, Novartis, outside the submitted work. Dr. Busse reports grants from Berlin University Alliance, non-financial support from German Federal Ministry of Health, during the conduct of the study.

### Data sharing statement

Data used in this study is publicly available as mentioned in reference [7] and [21].

## Appendix Supplementary materials

Image, application 1

Image, application 2

Image, application 3

Image, application 1

Image, application 2

Image, application 3
