# Supplementary material for: Different spreading dynamics throughout Germany during the second wave of the COVID-19 pandemic: a time series study based on national surveillance data
Source: Lancet Reg Health Eur. 2021 Jun 27;6:100151. doi: 10.1016/j.lanepe.2021.100151 (PMC8454815; doi:10.1016/j.lanepe.2021.100151)
Supplement: Supplementary file 1 [file mmc1.docx]

SUPPLEMENT

**Different spreading dynamics throughout Germany during the second wave of the COVID-19 pandemic: impact of public health interventions**

Prof. rer.nat Andreas Schuppert ^1#$^, Katja Polotzek, Dipl. Math. ^2$^, Prof. Dr. med. Jochen Schmitt, MPH ^2^, Prof. Reinhard Busse ^3^, Jens Karschau, PhD ^2*^, and Prof. Christian Karagiannidis, MD ^4*#^

SUPPEMENTARY TEXTS

**Smoothing of seven day reporting periodicity**

The original infection time series for age groups and federal states was decomposed into seven series, one for each day per week. Division of each weekday-specific time series by the ratio of the median of the weekday-specific infections between October and December 2020 and the median of infections throughout the respective time window before pooling again to a time series for all days reduced the weekly periodicity. The resulting time series were smoothed by outlier elimination and Savitzky-Golay filtering. To reduce remaining weekly periodicity, the change rates x’(t) were calculated as weekly change rates and smoothed by Savitzky-Golay filtering with bootstrapping.

SUPPEMENTARY FIGURES

**Supplemental Figure 1**

Strength of non-pharmaceutical interventions (NPI) during the second wave of the COVID-19 pandemic. The thickness of the coloured bars classifies the strength of NPI into four categories: no restriction (thinnest), weak (recommendations, little broad regulations), strong (specific stronger restrictions), entire ban (thickest). Solid bars denote measures on national level, transparent bars indicate measures on federal state or regional level aiming at control of local hotspots. In all areas of public life mild regulations like distance and mask requirements have been ongoing since the first wave in early 2020. Recommendations considered divided or remote teaching at schools and universities just as similarly for vocational activities. A partial shutdown from November 4, 2020 aimed at mainly leisure time and personal hygienics facilities and was extended by further restrictions starting from December 16, 2020, regarding the entire prevention of contacts in schools and retail shops.

**Supplemental Figure 2**

Change of weekly numbers of new incidences per 100 000 population by state cluster and age groups. For each of the three clusters of states (panel A, B, and C) and age groups (x-axes) the heights of the bars indicate the change of weekly new infections during the different periods of NPIs from October, 2020 to December, 2020. These characteristics highlight the similarities and differences of the infection dynamics in the state groups during each of the four phases (October growth, partial shutdown in November, December growth, extended shutdown from mid-December). The incidences and rates of change are overall lower in the northern group. The exponential growth (cp. Fig. 3) in October (blue) affects the age groups likewise across all states with highest increases among the working cohort of age 15-59. Partial shutdown regulations (orange) yielded negative growth rates in this cohort in the northern and southwestern but not in the eastern states. Among the group of age 60 or higher the growth rate remained positive across even all states. The December growth (yellow) affected all age groups in all states again. Negative weekly changes with decreasing weekly incidences were obtained by extended shutdown regulations (purple).

**Supplemental Figure 3**

Weekly number of PCR tests in Germany from August 2020, to December 2020. By November 3, 2020, the national test strategy was adapted to the autumn and winter seasons with more specific test criteria (cp. Fig. 1). Positive rapid antigen tests are doubled-check by additional PCR tests and further increase incidences when indicated. The rate of positive tests among all conducted PCR tests increased during the entire considered period of the second wave of infections.

**Supplemental Figure 4**

Proportion of age groups in ten-year bands per case incidences per week. With the beginning in October 2020 age groups of the generation older than 60 years, exhibited a constant increase every week. The age group of children's parents (20-39) showed a decline. The age group younger than 20 years meanders had a slower decrease than compared to their parental age group. The group 40-59 years had no clear trend.
