# Supplementary figures and images for: Different spreading dynamics throughout Germany during the second wave of the COVID-19 pandemic: a time series study based on national surveillance data
Source: Lancet Reg Health Eur. 2021 Jun 27;6:100151. doi: 10.1016/j.lanepe.2021.100151 (PMC8454815; doi:10.1016/j.lanepe.2021.100151)

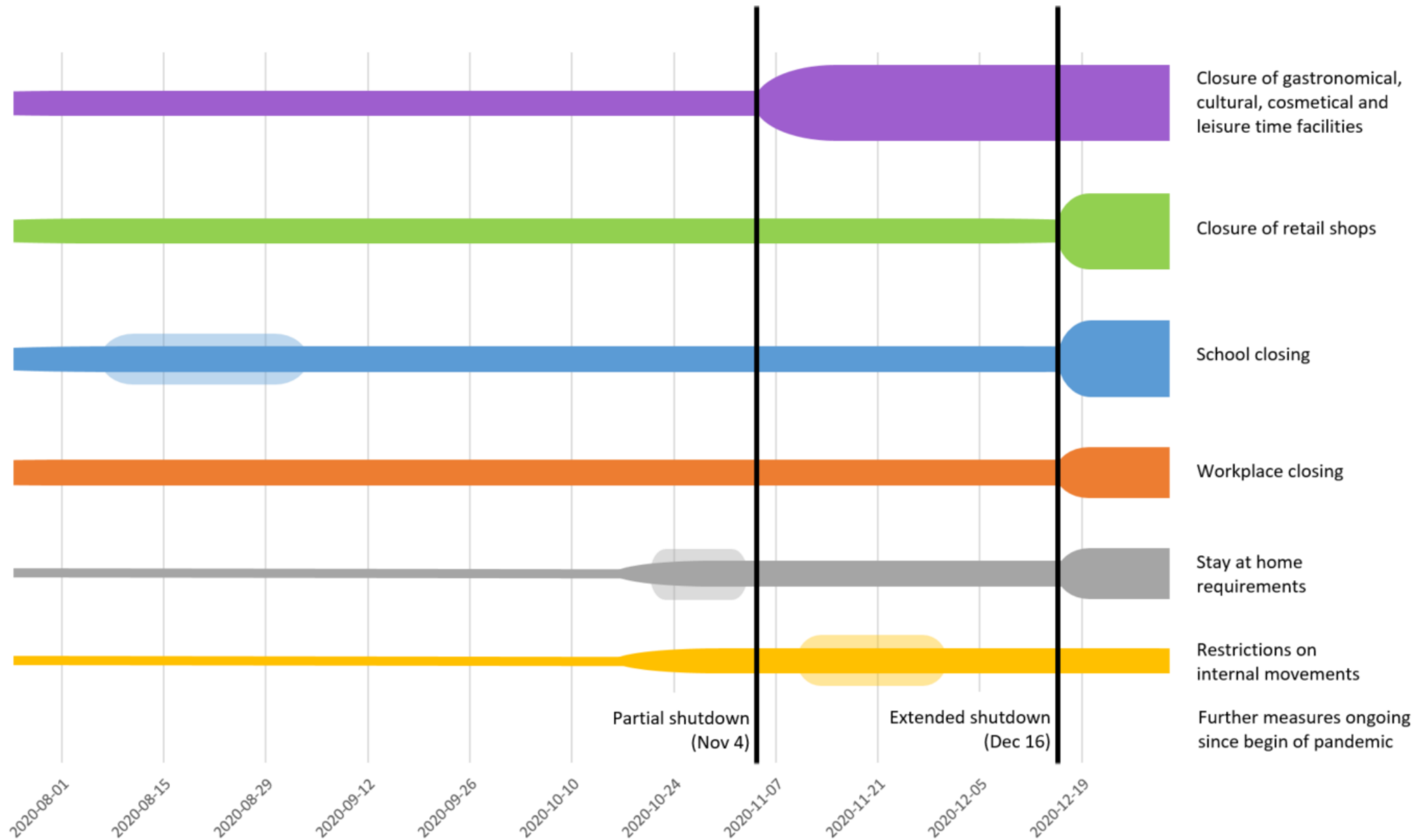

Supplement: Supplementary file 2 [file mmc2.pdf]

Supplemental Figure 2

A

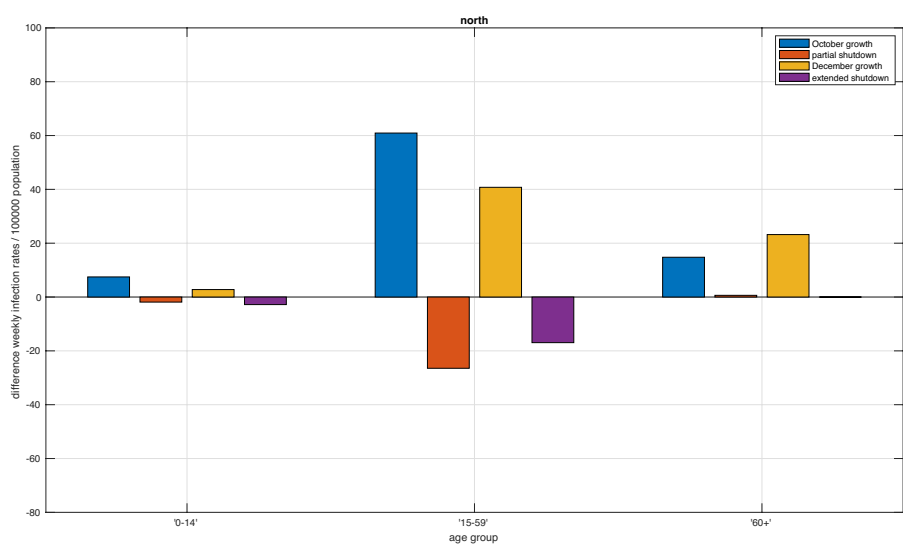

B

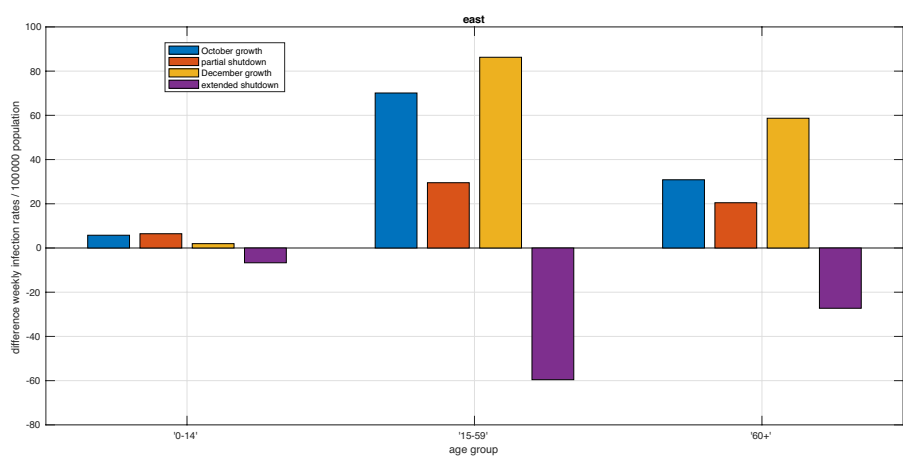

C

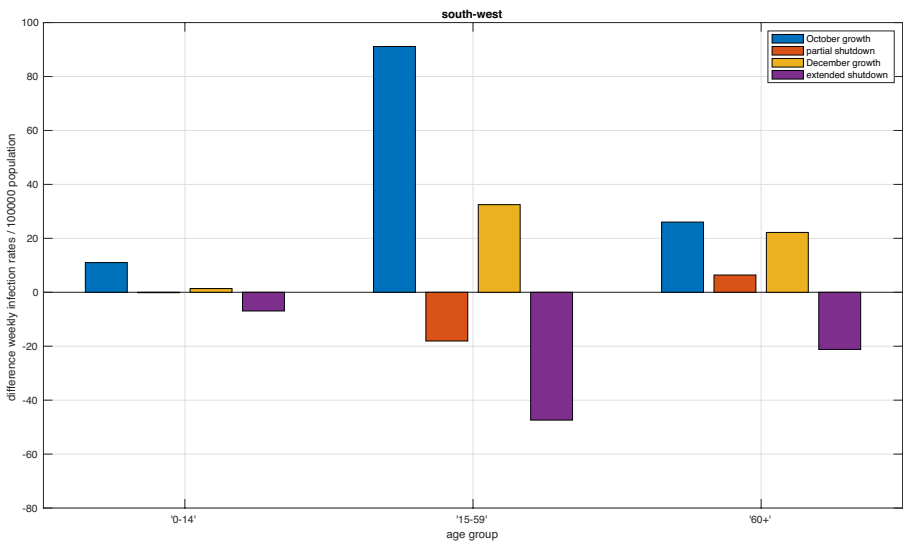

Supplement: Supplementary file 3 [file mmc3.pdf]

Supplemental Figure 3

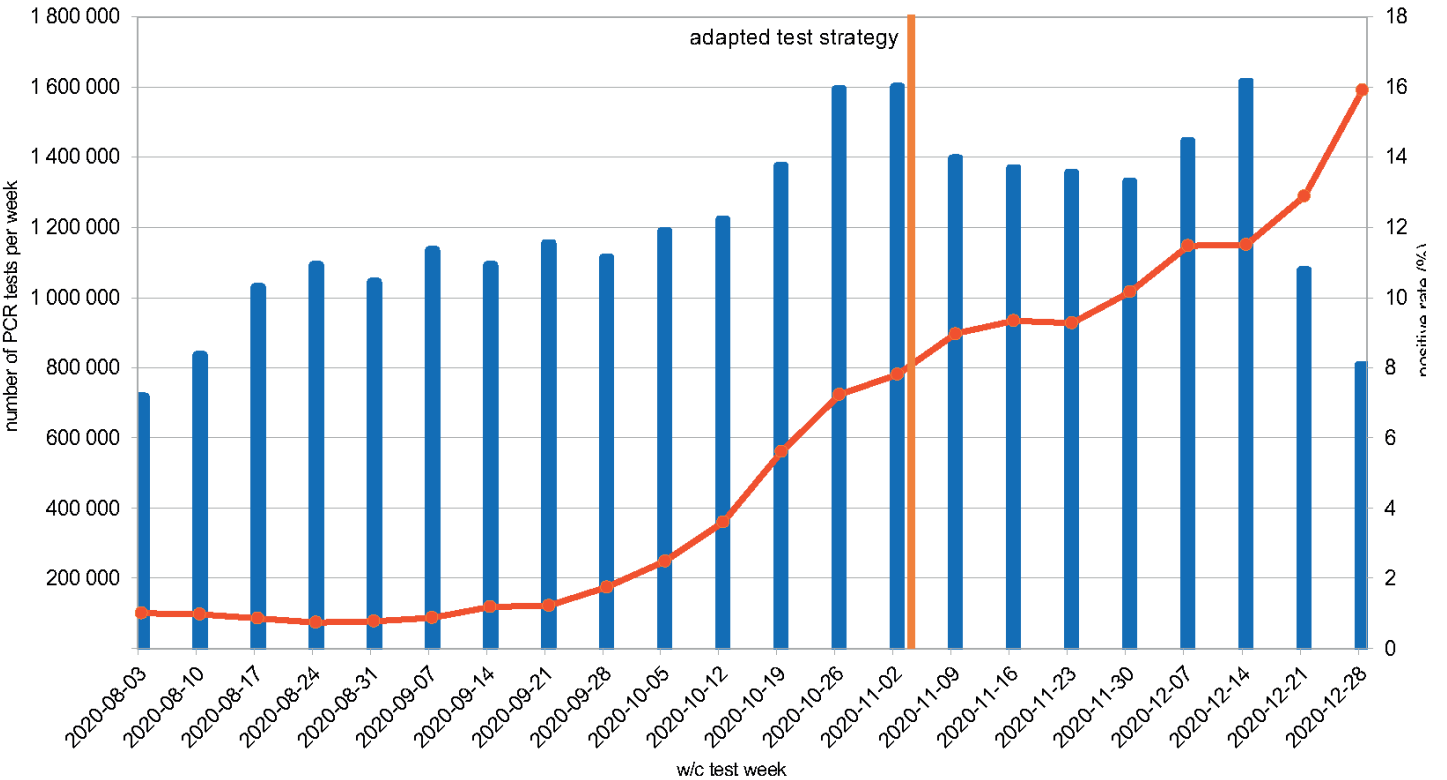

Supplement: Supplementary file 4 [file mmc4.pdf]

Supplemental Figure 4

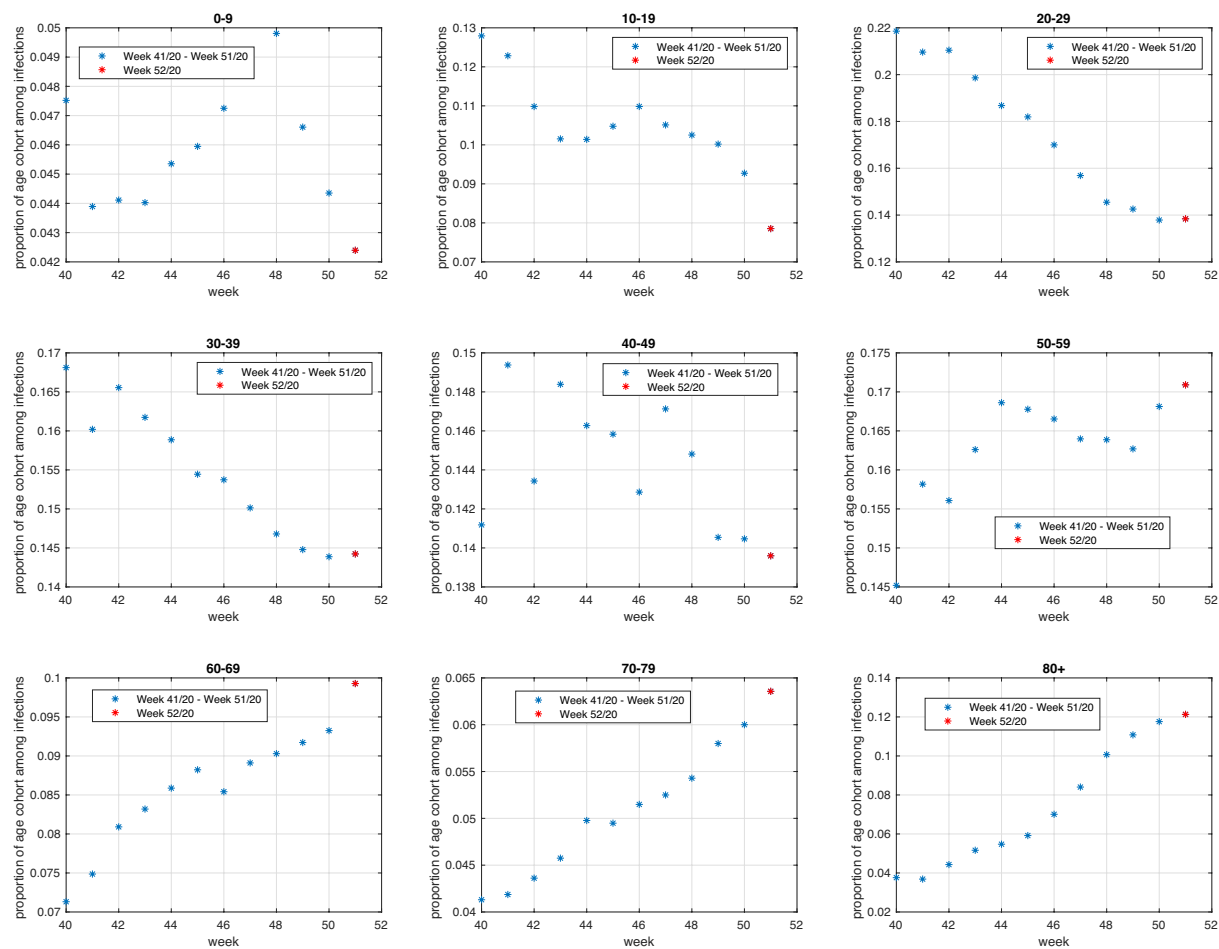

Supplement: Supplementary file 5 [file mmc5.pdf]

Supplemental Figure 5

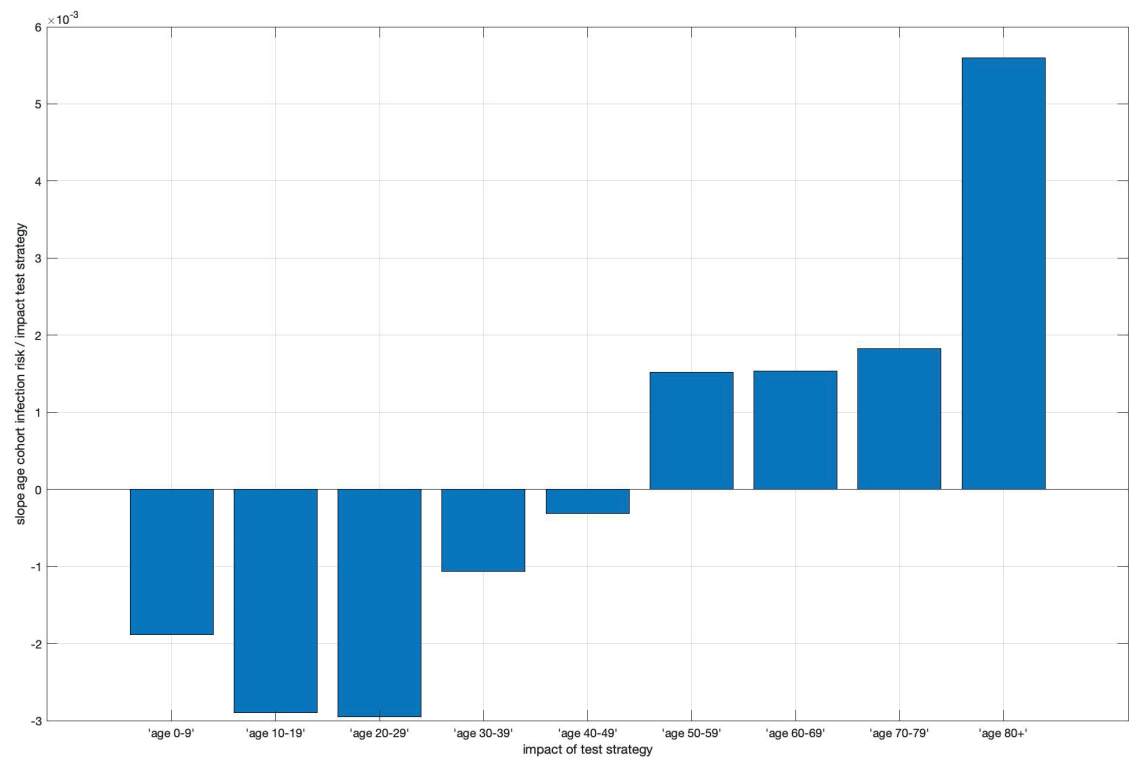

Supplement: Supplementary file 6 [file mmc6.pdf]
